# Association between the blood urea nitrogen-to-creatinine ratio and 3-month outcomes in patients with acute ischemic stroke: a secondary analysis based on a prospective cohort study

**DOI:** 10.3389/fneur.2024.1350116

**Published:** 2024-04-17

**Authors:** Hongjuan Liu, Yongjun Chen, Quan Zhou, Guixiang Guo, Bohong Hu, Fangchao Wan, Jun Wen

**Affiliations:** Changde Hospital, Xiangya School of Medicine, Central South University, Changde, China

**Keywords:** blood urea nitrogen, creatinine, stroke, adverse outcomes, cohort study

## Abstract

**Introduction:**

This study aimed to assess the correlation between the blood urea nitrogen (BUN)-to-creatinine (BUN/Cr) ratio and adverse outcomes (AOs) at 3 months in patients with acute ischemic stroke (AIS) in the Korean population.

**Methods:**

This cohort study encompassed 1906 cases of AIS at a South Korean hospital from January 2010 to December 2016. To determine the linear correlation between the BUN/Cr ratio and AOs in AIS, a binary logistic regression model (BLRM) was employed. Additionally, generalized additive models and techniques for smooth curve fitting were utilized to reveal the nonlinear dynamics between the BUN/Cr ratio and AOs in patients with AIS.

**Results:**

The prevalence of AOs was 28.65%, with a median BUN/Cr ratio of 18.96. Following adjustments for covariates, the BLRM disclosed that the association between the BUN/Cr ratio and the risk of AOs in patients with AIS did not attain statistical significance. Nevertheless, a nonlinear relationship surfaced, pinpointing an inflection point at 21.591. To the left of this inflection point, a 31.42% reduction in the risk of AOs was noted for every 1-unit surge in the Z score of the BUN/Cr ratio [odds ratio (OR) = 0.686, 95% confidence interval (CI): 0.519, 0.906, *p* = 0.008]. On the right side of the inflection point, the effect size (OR = 1.405, 95% CI: 1.018, 1.902, *p* = 0.039) was determined.

**Conclusion:**

The findings of this study underscore the intricate nature of the relationship between the BUN/Cr ratio and 3-month outcomes in patients with AIS, establishing a robust groundwork for future investigations.

## 1 Introduction

Stroke is a significant global health challenge; it is the second most prevalent cause of mortality and the third leading contributor to both death and disability worldwide (1, 2). Specifically, in 2019, ischemic stroke constituted 62.4% of all newly reported stroke cases (1). Regrettably, stroke imposes a heavy economic burden on the nation and society, which includes the cost of care required for patients with a poor prognosis after stroke and the direct or indirect loss of productivity. As the population continues to grow and age, the corresponding costs are expected to increase dramatically (2, 3). Accurate prognostication of the functional outcomes in patients with ischemic stroke assumes pivotal importance in tailoring individualized treatment regimens and optimizing post-stroke recovery dynamics. There are many factors affecting the prognosis of stroke, such as age, hypertension, diabetes, heart disease, and stroke etiology, which necessitate robust empirical substantiation. The intricate interplay between dehydration and post-stroke prognosis remains a subject of sustained scholarly deliberation, with divergent perspectives on the correlation between dehydration and unfavorable outcomes (4–6), counterpoised by dissenting opinions (7–9). In addition, the previous studies were almost all linear studies, with either a small sample size or the insufficient collection of indicators. This study was drawn from a substantial cohort of 2084 patients with AIS using a prospective registry spanning 6 years, ensuring a comprehensive representation of diverse cases. Biomarkers indicative of dehydration, notably the blood urea nitrogen/creatinine (BUN/Cr) ratio and plasma osmolality, have been scrutinized for their utility (10), with the present study opting for the former owing to its routine clinical applicability in contrast to the relatively less accessible plasma osmolality.

The primary objective of this research was to elucidate the nuanced relationship between the BUN/Cr ratio and AOs at the 3-month post-AIS juncture, thereby providing clinicians with nuanced insights into the optimal treatment protocol formulation in the realm of stroke care.

## 2 Materials and methods

### 2.1 Study design

This cohort investigation harnessed data spanning the temporal expanse from January 2010 to December 2016, which were meticulously attained from an exclusive prospective registry system centered in South Korea (11). The primary independent variable was the BUN/Cr ratio, while the dependent variable was the 3-month outcomes in cases with AIS, categorized as either AOs or favorable outcomes.

### 2.2 Data source

Information was obtained from the research conducted by Kang et al., titled “Geriatric nutritional risk index predicts AOs in AIS cases - automated undernutrition screen tool” (11). It is imperative to underscore the open-access nature of this article, governed by the Creative Commons Attribution License, thereby conferring unhindered permissions for utilization, dissemination, and reproduction across all media, contingent upon the stipulation of due credit to the original author and source (11).

### 2.3 Study population

**Figure 1:**
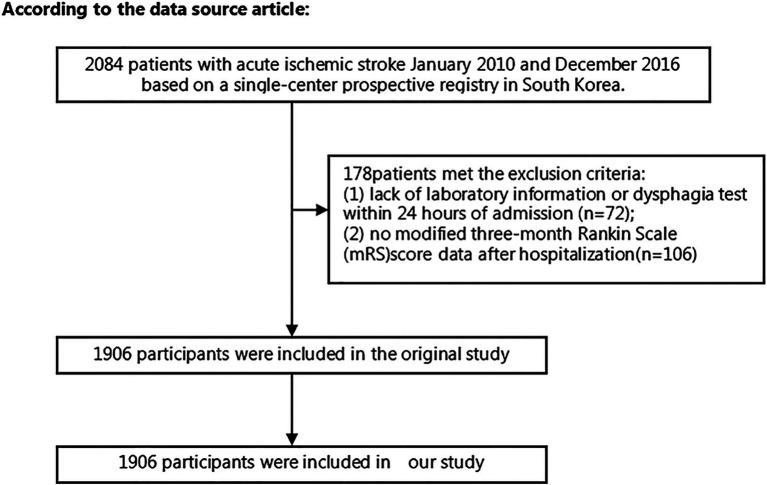
Flowchart of patient selection and exclusion in the original study.

Using data from a single-center prospective registry in South Korea that began enrollment in October 2002, we screened 2084 patients diagnosed with AIS who were admitted within 7 days of symptom onset from January 2010 to December 2016. The blood sample of each patient was presumed to be obtained from the first blood drawing on admission according to the hospital procedure. Strict exclusion criteria for the study included the following: (1) cases lacking dysphagia testing or relevant laboratory information within the first 24 h after admission; and (2) cases lacking of records of the modified Rankin scale (mRS) score at 3 months post-stroke. A comprehensive schematic detailing the intricate process of patient selection is presented in Figure 1. Given the antecedent approval by the Institutional Review Board of Seoul National University Hospital (Approval No. 1009–062-332) for the original study (11), no additional approval was required for the ensuing secondary analysis.

### 2.4 Variables

Derivation of the BUN/Cr ratio involved the division of the serum BUN concentration (mg/dl) by the serum Cr concentration (mg/dL), yielding a continuous variable. The subsequent categorization of the BUN/Cr ratio into quartiles was performed as follows: Q1: <13.846, Q2: 13.861–17.000, Q3: 17.007–21.311, and Q4: ≥21.333.

### 2.5 Three-month outcomes of patients with AIS

Assessment of the 3-month outcomes following the onset of AIS was executed through the utilization of the mRS score (12). The meticulous acquisition of data was performed via outpatient visits or methodically conducted structured telephone interviews (11). Participants underwent categorization into two distinct groups based on their outcomes: favorable outcomes, denoted by an mRS score ≤ 2, and AOs, characterized by an mRS score ≥ 3 (12).

### 2.6 Covariates

In the paradigm of our study, the covariates were judiciously selected, employing the Empower Stats’ Covariates module in tandem with our cumulative clinical acumen. This comprehensive selection encompassed a multitude of both continuous and categorical factors. Among the continuum were a diverse set of variables, including but not limited to the white blood cell (WBC) count, red blood cell (RBC) count, mean corpuscular hemoglobin concentration (MCHC), red blood cell distribution width (RDW), platelet (PLT) count, hemoglobin (HGB) level, hematocrit (HCT), mean corpuscular volume (MCV), total cholesterol (TC), serum triglyceride (TG) levels, low-density lipoproteins cholesterol (LDL-C) levels, high-density lipoproteins cholesterol (HDL-C) levels, alanine aminotransferase (ALT), aspartate aminotransferase (AST), BUN, Cr, glomerular filtration rate (GFR), serum albumin (ALB), total protein (TP), fasting blood glucose (FBG), activated partial thromboplastin time (APTT), body mass index (BMI), and fibrinogen (FIB). Concurrently, the categorical variables covered a broad spectrum, including age, sex, diabetes mellitus (DM), smoking habits, coronary heart disease (CHD), atrial fibrillation (AF), history of previous stroke/transient ischemic attack (TIA), hypertension, stroke etiology, and the National Institute of Health Stroke Scale (NIHSS) score (13). The meticulous retrieval of laboratory data within the initial 24 h of admission was obtained from the electronic medical records (11). The computation of BMI involved the division of weight in kilograms by the square of the height in meters (kg/m^2^).

### 2.7 Missing data processing

In the current investigation, the instances of missing data for key variables, namely TC, TG, HDL-C, LDL-C, FIB, and FBG, were 1 (0.00%), 107 (5.61%), 99 (5.19%), 75 (3.93%), 22 (1.15%), and 139 (7.29%), respectively. To mitigate the potential ramifications of these missing covariates on statistical efficiency during the modeling process, the study employed mean imputations.

### 2.8 Statistical analysis

The methodology for characterizing continuous variables involved the application of descriptive statistics, specifically, the mean ± standard deviation (SD) for variables exhibiting a Gaussian distribution, and the median (interquartile ranges) for those displaying a skewed distribution. Categorical variables were delineated through frequencies and percentages. The analytical framework encompassed the χ^2^, one-way analysis of variance (ANOVA), or Kruskal–Wallis H tests to discern differences across distinct BUN/Cr ratio groups. Subsequent to a rigorous collinearity assessment [Supplementary Table S1, selection method: the variable inflation factor (VIF) was calculated for each variable, if the highest VIF value ≥5, the variable was removed. The above step was repeated until all remaining variables had a VIF <5, resulting in the elimination of HGB, HCT, and TC], four nuanced models were constructed. These encompassed both univariate and multivariate binary logistic regression analyses, probing the intricate association between the BUN/Cr ratio and AOs in patients with AIS. The fourfold model structure comprised an unadjusted model, a minimally adjusted model accounting only for sociodemographic variables (age sex), a moderately adjusted model accounting for sociodemographic variables and some clinical features (smoking, BMI and past history), and a fully adjusted model accounting for sociodemographic variables and more clinical features (WBC, RBC, MCHC, RDW, AST, ALT, FBG, FIB, TG, LDL-C, ALB, TP, GFR, BMI, DM, previous stroke or TIA, hypertension, AF, CHD, stroke etiology, smoking, and NIHSS score); when latter variables were added to this model, the odds ratio (OR) of the matches changed by at least 10%. Effect sizes, elucidated with 95% confidence intervals (95% CIs), were meticulously documented, with adjustments informed by a synthesis of clinical expertise, literature findings, insights from the Empower Stats’ Covariates module, and outcomes derived from the univariate analyses. In the pursuit of result robustness, a sensitivity analysis was performed by categorizing the BUN/Cr ratio into quartiles and assessing the *p*-value for trend. We opted to exclude cases with specific conditions, such as DM, abnormal FBG, hypercholesterolemia, elevated BMI, and renal failure. Potential unmeasured confounding factors were systematically explored utilizing E-values. The examination of potential nonlinearity in the relationship between the BUN/Cr ratio and AOs employed sophisticated techniques, including application of generalized additive models (GAMs) and the employment of intricate smooth curve fitting methods, such as penalized splines. The detection of nonlinearity prompted the initiation of a recursive algorithm to pinpoint the inflection point. Subsequently, this led to the establishment of a two-piece binary logistic regression model (BLRM), strategically deployed on either side of this pivotal juncture. The most appropriate model was adjudged based on a log-likelihood ratio test.

All reported findings strictly adhered to the STROBE statement, and the statistical analysis encompassed the usage of both R and Empower Stats (X&Y Solutions, Inc., Boston, MA, United States) software. Significance, in this context, was ascribed to a two-tailed *p* < 0.05 (14).

## 3 Results

### 3.1 Participants’ characteristics

**Figure 2:**
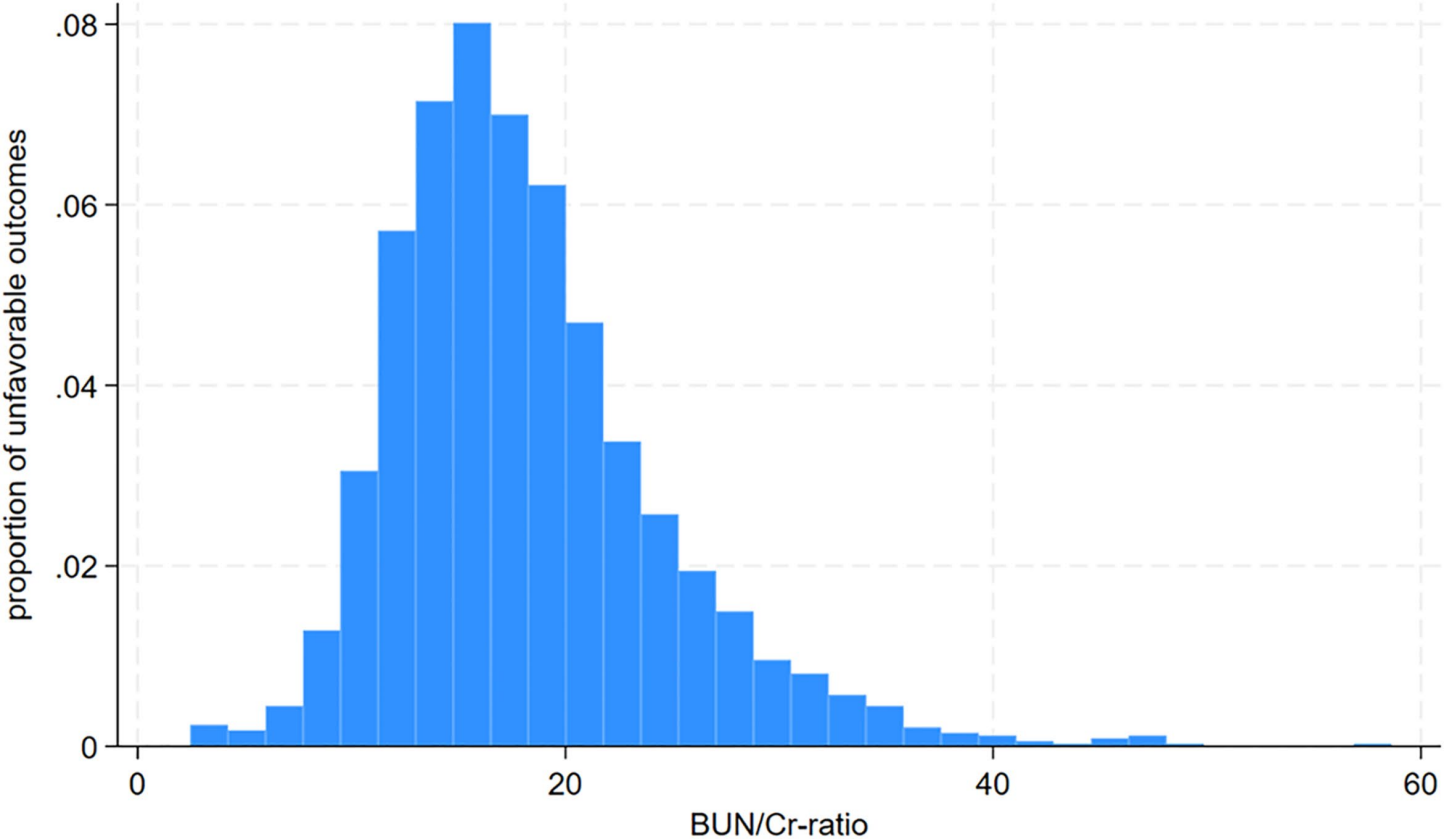
Distribution and characteristics of participants stratified by the blood urea nitrogen/creatinine ratio (BUN/Cr ratio) quartiles.

Following screening with strict inclusion and exclusion criteria, this study excluded cases lacking dysphagia testing or relevant laboratory information within 24 h after admission (*n* = 72) and cases lacking a mRS score at 3 months after stroke (*n* = 106). After the exclusion of 178 cases, the final analysis cohort consisted of 1906 individuals. Table 1 and Supplementary Table S2 delineate the demographic and clinical attributes of the study cohort. The analysis cohort was 61.28% male (1,168 individuals). Participants were stratified into age categories: <60 years (436 participants, 22.88%), 60 to <70 years (505 participants, 26.50%), 70 to <80 years (670 participants, 35.15%), and ≥ 80 years (295 participants, 15.48%). The breakdown of the stroke etiology indicated that 606 (31.79%), 365 (19.15%), 493 (25.87%), 171 (8.97%), and 271 (14.22%) cases were small-vessel occlusion (SVO), large-artery atherosclerosis (LAA), cardiogenic embolism (CE), other determined, and undetermined, respectively. The median (interquartile ranges) of the NIHSS score was 5.0 (1.0–11.0). Participants were further categorized into subgroups based on BUN/Cr ratio quartiles: Q1: <13.846, Q2: 13.861–17.000, Q3: 17.007–21.311, and Q4: ≥21.333. Compared with Q1, Q4 exhibited elevated values of HDL-c, BUN, and GFR, with reduced levels of TG, Cr, and FIB. Q4 featured a higher proportion of women (61.33%), individuals with hypertension (65.90%), and DM cases (34.72%). The distribution of the ratio is visually depicted in Figure 2, illustrating a skewed distribution ranging from 2.469 to 58.621, with a median of 18.163. Supplementary Table S3 shows participants’ baseline characteristics classified by functional outcomes.

**Table 1:** Participants’ baseline characteristics categorized by the quartiles of the BUN/Cr ratio.

Characteristics	BUN/Cr ratio				*P*
	Q1 (2.469–13.846)	Q2 (13.861–17)	Q3 (17.007–21.311)	Q4 (21.333–58.621)	
Number of participants	477	476	472	481	
Demographics					
Age, years					<0.001
<60	149 (31.237%)	119 (25%)	94 (19.915%)	74 (15.385%)	
60–70	120 (25.157%)	119 (25%)	135 (28.602%)	131 (27.235%)	
70–80	151 (31.656%)	173 (36.345%)	162 (34.322%)	184 (38.254%)	
≥80	57 (11.950%)	65 (13.655%)	81 (17.161%)	92 (19.127%)	
Gender, n(%)					<0.001
Male	359 (75.262%)	328 (68.908%)	295 (62.5%)	186 (38.669%)	
Female	118 (24.738%)	148 (31.092%)	177 (37.5%)	295 (61.331%)	
Smoking, n(%)	222 (46.541%)	208 (43.697%)	185 (39.195%)	135 (28.067%)	<0.001
Hypertension	292 (61.216%)	293 (61.555%)	309 (65.466%)	317 (65.904%)	0.279
DM	137 (28.721%)	152 (31.933%)	158 (33.475%)	167 (34.719%)	0.221
CHD	60 (12.579%)	52 (10.924%)	56 (11.864%)	52 (10.811%)	0.806
AF	93 (19.497%)	97 (20.378%)	126 (26.695%)	91 (18.919%)	0.012
Previous stroke/TIA	99 (20.755%)	96 (20.168%)	106 (22.458%)	101 (20.998%)	0.848
Clinical features					
BMI (kg/m^2^)	23.49 (21.42–25.56)	23.580 (21.527–25.835)	23.485 (21.36–25.543)	23.07 (21.05–24.95)	0.052
Baseline NIHSS score					0.017
<6	352 (73.795%)	364 (76.471%)	343 (72.669%)	318 (66.112%)	
6–13	77 (16.143%)	68 (14.286%)	72 (15.254%)	91 (18.919%)	
≥14	48 (10.063%)	44 (9.244%)	57 (12.076%)	72 (14.969%)	
TC (mg/dL)	173 (146–199)	179 (150.75–212)	176 (148.75–202)	182 (154–211)	0.020
TG (mg/dL)	105.338 (83–135)	100 (77.75–136.25)	96 (73.75–127)	95 (72–124)	<0.001
HDL-C (mg/dL)	43 (35–50)	44.165 (37–54)	45 (38–55)	46 (40–57)	<0.001
LDL-C (mg/dL)	104.155 (82–129)	104.155 (83–131.25)	104.155 (82.75–126.25)	105 (86–133)	0.499
BUN (mg/dL)	12 (10–15)	14 (12–17)	17 (14–20)	20 (17–24)	<0.001
Cr (mg/dL)	1.02 (0.85–1.24)	0.93 (0.79–1.113)	0.875 (0.74–1.04)	0.74 (0.63–0.91)	<0.001
GFR (%)	70.5 (55.5–87.4)	75.45 (59.75–89.)	78.6 (64.68–93.2)	86.8 (72.5–101.8)	<0.001
ALT (U/L)	17 (13–25)	18 (14–26)	19 (13.75–27)	18 (13–26)	0.156
AST (U/L)	23 (18–29)	24 (19–29.25)	23 (19–30)	22 (18–29)	0.150
ALB (g/dl)	4.1 (3.8–4.3)	4.1 (3.9–4.4)	4.1 (3.8–4.3)	4.0 (3.8–4.3)	0.002
TP (g/dl)	7.0 (6.7–7.4)	7.1 (6.8–7.5)	7.0 (6.6–7.4)	7.0 (6.6–7.4)	0.045
FBG (mg/dl)	98 (86–109)	98 (86–111)	99.055 (86.75–118)	99.055 (90–119)	0.003
Ischemic stroke subtype					0.043
SVO	153 (32.075%)	162 (34.034%)	143 (30.297%)	148 (30.769%)	
LAA	86 (18.029%)	100 (21.008%)	82 (17.373%)	97 (20.166%)	
CE	104 (21.803%)	119 (25%)	148 (31.356%)	122 (25.364%)	
Other determined	56 (11.740%)	35 (7.353%)	35 (7.415%)	45 (9.356%)	
Undetermined	78 (16.352%)	60 (12.605%)	64 (13.559%)	69 (14.345%)	
Unfavorable outcome (%)	139 (29.140%)	122 (25.630%)	121 (25.636%)	164 (34.096%)	0.010

Values are mean ± standard deviation, median (quartile), or number (%). BUN/Cr ratio, blood urea nitrogen to creatinine ratio; TG, triglyceride; TC, total cholesterol; HDL-C, high-density lipoprotein cholesterol; LDL-C, low-density lipoproteins cholesterol; BUN, blood urea nitrogen; Cr, serum creatinine; GFR, glomerular filtration rate; ALT, alanine aminotransferase; AST, aspartate aminotransferase; ALB, serum albumin; TP, total protein; FBG, fasting blood glucose; BMI, body mass index; DM, diabetes mellitus; CHD, coronary heart disease; AF, atrial fibrillation; TIA, transient ischemia attack; LAA, large-artery atherosclerosis; SVO, small-vessel occlusion; CE, cardio embolism; NIHSS, National institute of health stroke scale.

### 3.2 Incidence rate 3-month AOs in patients with AIS

A total of 546 participants encountered AOs, yielding an overall incidence rate of 28.65% (26.65–30.71%) (Table 2). The specific incidence rates across the BUN/Cr ratio quartiles were: Q1, 29.14% (25.19–33.34%); Q2, 25.63% (21.86–29.70%); Q3, 25.64% (21.85–29.72%); and Q4, 34.10% (29.96–38.42%).

**Table 2:** Incidence rate of adverse outcomes at 3 months following the occurrence of stroke.

BUN/Cr ratio	Participants (n)	Unfavorable outcome events (mRS score ≥ 3)	Incidence of unfavorable outcomes (%) (95% CI)
Total	1906	546	28.65 (26.65–30.71)
Q1 (2.469–13.846)	477	139	29.14 (25.19–33.34)
Q2 (13.861–17.000)	476	122	25.63 (21.86–29.70)
Q3 (17.007–21.311)	472	121	25.64 (21.85–29.72)
Q4 (21.333–58.621)	481	164	34.10 (29.96–38.42)

BUN/Cr ratio, blood urea nitrogen to creatinine ratio; mRS, modifed Rankin scale.

### 3.3 Results of the univariate analysis using the BLRM

The univariate analysis elucidated that AOs in patients with AIS lacked statistically significant associations with the MCV (OR = 0.988, 95% CI: 0.969, 1.007), PLT (OR = 0.999, 95% CI: 0.998, 1.001), HDL-C (OR = 0.997, 95% CI: 0.990, 1.005), Cr (OR = 1.017, 95% CI: 0.926, 1.116), GFR (OR = 1.000, 95% CI: 0.996, 1.004), and APTT (OR = 0.988, 95% CI: 0.970, 1.007) (all *p* > 0.05). Conversely, a positive association was found with the WBC (OR = 1.079, 95% CI: 1.043, 1.116), RDW (OR = 1.232, 95% CI: 1.154, 1.316), BUN (OR = 1.016, 95% CI: 1.006, 1.027), ALT (OR = 1.009, 95% CI: 1.002, 1.016), FBG (OR = 1.008, 95% CI: 1.005, 1.010), FIB (OR = 1.003, 95% CI: 1.002, 1.004), and BUN/Cr ratio (OR = 1.027, 95% CI: 1.012, 1.043) (all *p* < 0.05). Furthermore, female participants (OR = 1.660, 95% CI: 1.357, 2.030), those aged ≥80 years (OR = 3.996, 95% CI: 2.874, 5.557), individuals with hypertension (OR = 1.343, 95% CI: 1.088, 1.658), diabetes mellitus (OR = 1.446, 95% CI: 1.174, 1.781), a history of previous stroke/TIA (OR = 1.811, 95% CI: 1.437, 2.283), an NIHSS score ≥ 14 (OR = 15.106, 95% CI: 10.827, 21.075), atrial fibrillation (AF) (OR = 2.001 [1.590, 2.517]), and other determined stroke etiologies (OR = 2.073, 95% CI: 1.458, 2.928) showed an elevated risk of AOs (all *p* < 0.05). In contrast, RBC (OR = 0.583, 95% CI: 0.498, 0.682), HGB (OR = 0.819, 95% CI: 0.779, 0.862), HCT (OR = 0.932, 95% CI: 0.916, 0.949), MCHC (OR = 0.868, 95% CI: 0.797, 0.947), TC (OR = 0.995, 95% CI: 0.993, 0.998), TG (OR = 0.996, 95% CI: 0.994, 0.998), LDL-C (OR = 0.997, 95% CI: 0.994, 1.000), AST (OR = 0.993, 95% CI: 0.986, 1.000), ALB (OR = 0.274, 95% CI: 0.215, 0.350), TP (OR = 0.674, 95% CI: 0.573, 0.793), and BMI (OR = 0.915, 95% CI: 0.886, 0.945) exhibited negative associations with the risk of AOs (Table 3; Supplementary Table S4).

**Table 3:** Determinants of adverse outcomes in AIS assessed through the univariate regression analysis.

Characteristics	OR (95% CI)	*P*
Demographics		
Age, years		
<60	Ref.	
60–70	1.153 (0.839, 1.586)	0.380
70–80	1.900 (1.423, 2.536)	<0.001
≥80	3.996 (2.874, 5.557)	<0.001
Gender, n(%)		
Male	Ref.	
Female	1.660 (1.357, 2.030)	<0.001
Smoking, n(%)		
No	Ref.	
Yes	0.608 (0.493, 0.751)	<0.001
Hypertension		
No	Ref.	
Yes	1.343 (1.088, 1.658)	0.006
DM		
No	Ref.	
Yes	1.446 (1.174, 1.781)	0.001
CHD		
No	Ref.	
Yes	1.025 (0.752, 1.397)	0.877
AF		
No	Ref.	
Yes	2.001 (1.590, 2.517)	<0.001
Previous stroke/TIA		
No	Ref.	
Yes	1.811 (1.437, 2.283)	<0.001
Clinical features		
BMI (kg/m^2^)	0.915 (0.886, 0.945)	<0.001
Baseline NIHSS score		
<6	ref.	
6–13	6.366 (4.874, 8.315)	<0.001
≥14	15.106 (10.827, 21.075)	<0.001
TC (mg/dL)	0.995 (0.993, 0.998)	<0.001
TG (mg/dL)	0.996 (0.994, 0.998)	<0.001
HDL-C (mg/dL)	0.997 (0.990, 1.005)	0.459
LDL-C (mg/dL)	0.997 (0.994, 1.000)	0.024
BUN (mg/dL)	1.016 (1.006, 1.027)	0.003
Cr (mg/dL)	1.017 (0.926, 1.116)	0.732
GFR (%)	1.000 (0.996, 1.004)	0.961
ALT (U/L)	1.009 (1.002, 1.016)	0.009
AST (U/L)	0.993 (0.986, 1.000)	0.041
ALB (g/dL)	0.274 (0.215, 0.350)	<0.001
TP (g/dL)	0.674 (0.573, 0.793)	<0.001
FBG (mg/dL)	1.008 (1.005, 1.010)	<0.001
BUN/Cr ratio	1.027 (1.012, 1.043)	0.001
Ischemic stroke subtype		
SVO	Ref.	
LAA	0.622 (0.452, 0.856)	0.004
CE	1.496 (1.156, 1.935)	0.002
Other determined	2.073 (1.458, 2.948)	<0.001
Undetermined	0.875 (0.629, 1.218)	0.428

BUN/Cr ratio, blood urea nitrogen to creatinine ratio; TG, triglyceride; TC, total cholesterol; HDL-C, high-density lipoprotein cholesterol; LDL-C, low-density lipoproteins cholesterol; BUN, blood urea nitrogen; Cr, serum creatinine; GFR, glomerular filtration rate; ALT, alanine aminotransferase; AST, aspartate aminotransferase; ALB, serum albumin; TP, total protein; FBG, fasting blood glucose; BMI, body mass index; DM, diabetes mellitus; CHD, coronary heart disease; AF, atrial fibrillation; TIA, transient ischemia attack; LAA, large-artery atherosclerosis; SVO, small-vessel occlusion; CE, cardio embolism; NIHSS, national institute of health stroke scale.

### 3.4 Results of the multivariate logistic regression analysis using the BLRM

Four distinct models were constructed utilizing the BLRM to thoroughly explore the association between the BUN/Cr ratio and the likelihood of AOs in patients with AIS. The unadjusted model revealed a 2.7% rise in the risk of AOs for every 1-unit increase in the BUN/Cr ratio (OR = 1.027, 95% CI: 1.012, 1.043, *p* = 0.001), signifying statistical significance. The moderately adjusted model revealed a 1.7% rise in the risk of AOs for every 1-unit increase in the BUN/Cr ratio (OR = 1.017, 95% CI: 1.001, 1.034, *p* = 0.043), showing statistical significance. However, both the minimally adjusted and fully adjusted models in the multivariate logistic regression analysis did not yield statistically significant results (all *p* > 0.05) (Table 4).

**Table 4:** Examination of the intricate relationship between the BUN/Cr ratio and adverse outcomes 3 months post-stroke through diverse analytical models.

Variable	Crude model (OR, 95% CI)	*P*	Model I (OR, 95% CI)	*P*	Model II (OR, 95% CI)	*P*	Model III (OR, 95%CI)	*P*
BUN/Cr ratio	1.027 (1.012, 1.043)	0.001	1.013 (0.996, 1.030)	0.124	1.017 (1.001, 1.034)	0.043	0.994 (0.973, 1.015)	0.569
BUN/Cr ratio (quartiles)								
Q1 (2.469–13.846)	ref.		ref.		ref.		ref.	
Q2 (13.861–17.000)	0.838 (0.630, 1.115)	0.225	0.770 (0.574, 1.032)	0.081	0.803 (0.597, 1.079)	0.145	0.814 (0.572, 1.157)	0.251
Q3 (17.007–21.311)	0.838 (0.630, 1.116)	0.226	0.723 (0.537, 0.972)	0.032	0.713 (0.529, 0.960)	0.026	0.609 (0.424, 0.876)	0.008
Q4 (21.333–58.621)	1.258 (0.957, 1.653)	0.099	0.973 (0.726, 1.304)	0.854	1.032 (0.770, 1.384)	0.831	0.702 (0.484, 1.019)	0.063
P for trend		0.105		0.787		0.988		0.027

Crude Mode1: We did not adjust for any covariates. Model I: We adjusted age and sex. Model II: We adjusted age, sex, smoking, BMI, DM, previous stroke or TIA, hypertension, AF, and CHD. Model III: We adjusted age, sex, WBC, RBC, MCHC, RDW, AST, ALT, FBG, FIB, TG, LDL-C, ALB, TP, GFR, BMI, DM, previous stroke or TIA, hypertension, AF, CHD, stroke etiology, smoking, and NIHSS score.

### 3.5 Sensitivity analysis

Conducting a sensitivity analysis entailed the initial transformation of the BUN/Cr ratio from a continuous variable to a categorical variable, employing quartiles, and subsequently reintegrating the categorically transformed BUN/Cr ratio into the model. The multivariate-adjusted model disclosed an inverse association, presenting an OR of 0.609 (95% CI: 0.424, 0.876) for the Q3 versus the Q1. However, Q4, with an OR of 0.702 (95% CI: 0.484, 1.019), did not achieve statistical significance (Table 4, Model III). This observation suggests the potential existence of a nonlinear relationship between the BUN/Cr ratio and AOs in patients with AIS. Furthermore, an E-value of 1.88 surpassed the relative risk (RR; 1.54) associated with unmeasured confounders, indicating that unknown or unmeasured confounders had a minimal influence on the association between the BUN/Cr ratio and the likelihood of AOs in patients with AIS.

In an additional iteration of the sensitivity analysis, cases with TC ≥200 mg/dL, DM, FBG ≥6.1 mmol/L, BMI ≥25 kg/m^2^, and Cr1.2 mg/dL were selectively excluded. The findings unveiled that the previously observed linear relationship between the BUN/Cr ratio and the risk of AOs remained statistically insignificant following meticulous adjustments for confounding factors (all *p* > 0.05) (Table 5). Consequently, the purported linear association between the BUN/Cr ratio and the risk of AOs did not hold true, both in the overall population and within the specific subgroups. The sensitivity analysis accounted for all covariates, including age, sex, WBC, RBC, MCHC, RDW, AST, ALT, FBG, FIB, TG, LDL-C, ALB, TP, GFR, BMI, DM, history of stroke or TIA, hypertension, AF, CHD, stroke etiology, smoking, and the NIHSS score. Notably, Model I for non-DM cases did not include DM as an adjusted covariate.

**Table 5:** Exploration of the correlation between the BUN/Cr ratio and adverse outcomes.

BUN/Cr ratio	OR (95%CI)	*P*
Model I	0.994 (0.969, 1.019)	0.622
Model II	0.992 (0.968, 1.015)	0.483
Model III	0.995 (0.971, 1.019)	0.662
Model IV	0.994 (0.969, 1.020)	0.649
Model V	0.993 (0.971, 1.016)	0.559

Model I was the sensitivity analysis in participants without DM. We adjusted for age, sex, WBC, RBC, MCHC, RDW, AST, ALT, FBG, FIB, TG, LDL-C, ALB, TP, GFR, BMI, previous stroke or TIA, hypertension, AF, CHD, stroke etiology, smoking, and NIHSS score. Model II was the sensitivity analysis in participants without BMI ≥ 25 kg/m^2^. We adjusted age, sex, WBC, RBC, MCHC, RDW, AST, ALT, FBG, FIB, TG, LDL-C, ALB, TP, GFR, BMI, DM, previous stroke or TIA, hypertension, AF, CHD, stroke etiology, smoking, and NIHSS score. Model III was the sensitivity analysis in participants without TC ≥ 200 mg/L. We adjusted age, sex, WBC, RBC, MCHC, RDW, AST, ALT, FBG, FIB, TG, LDL-C, ALB, TP, GFR, BMI, DM, previous stroke or TIA, hypertension, AF, CHD, stroke etiology, smoking, and NIHSS score. Model IV was the sensitivity analysis in participants without FBG ≥ 6.1 mmol/L. We adjusted age, sex, WBC, RBC, MCHC, RDW, AST, ALT, FBG, FIB, TG, LDL-C, ALB, TP, GFR, BMI, DM, previous stroke or TIA, hypertension, AF, CHD, stroke etiology, smoking, and NIHSS score. Model V was the sensitivity analysis in participants without Cr > 1.2 mg/dL. We adjusted age, sex, WBC, RBC, MCHC, RDW, AST, ALT, FBG, FIB, TG, LDL-C, ALB, TP, GFR, BMI, DM, previous stroke or TIA, hypertension, AF, CHD, stroke etiology, smoking, and NIHSS score. OR, odds ratios; CI, confidence; Ref, reference; BUN/Cr ratio, blood urea nitrogen/creatinine ratio.

In addition, we performed a subgroup analysis based on the TOAST subtypes and different HDL-C levels; however, the results of the analysis were not statistically significant (Supplementary Table S5).

### 3.6 Nonlinearity was addressed by the GAM

**Figure 3:**
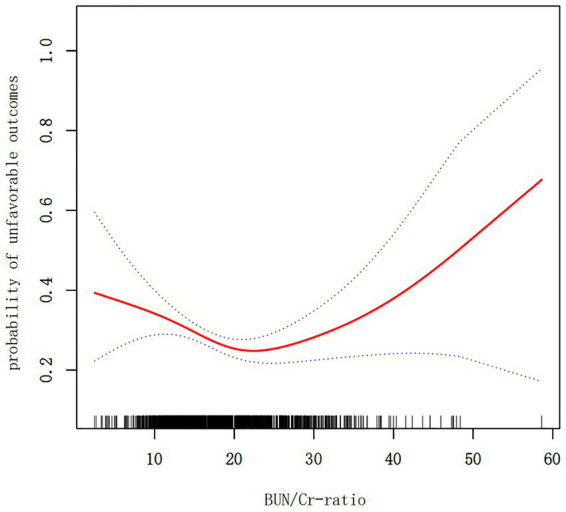
A nonlinear U-shaped relationship was observed between the blood urea nitrogen/creatinine ratio (BUN/Cr ratio) and unfavorable outcomes in patients with acute ischemic stroke (AIS).

The outcomes derived from the multivariate BLRM did not achieve statistical significance, suggesting the potential influence of intricate nonlinear relationships. Furthermore, the investigation into multivariate-adjusted models utilizing BUN/Cr ratio quartiles as categorical variables hinted at a complex nonlinear association between the BUN/Cr ratio and AOs in patients with AIS. Through the employment of sophisticated statistical techniques, such as GAM and smooth curve fitting, while adjusting for a comprehensive array of covariates, including age, sex, WBC, RBC, MCHC, RDW, AST, ALT, FBG, FIB, TG, LDL-C, ALB, TP, GFR, BMI, DM, history of stroke or TIA, hypertension, AF, CHD, stroke etiology, smoking, and the NIHSS score, a distinctive U-shaped relationship was revealed between the BUN/Cr ratio and the risk of AOs in patients with AIS (Figure 3). To comprehensively capture the nuances of this relationship, a piecewise BLRM was deployed, accommodating two distinctive slopes, with model selection predicated on the log-likelihood ratio test in the sensitivity analysis. Importantly, the *p*-value for the log-likelihood ratio test in this study was <0.05. Utilizing a recursive algorithm, the inflection point was identified at 21.591; the effect sizes and CIs were meticulously computed on both sides of this inflection point using the two-piecewise BLRM. Notably, on the left side of the inflection point, each 1-unit increment in the Z score of the BUN/Cr ratio was associated with a 31.4% lower risk of AOs (OR = 0.686, 95% CI: 0.519, 0.906, *p* = 0.008). Conversely, on the right side of the inflection point, the effect size (OR) was 1.406 (95% CI: 1.018, 1.942, *p* = 0.039) (Table 6). In the analysis, a selective measure was implemented by excluding participants with a TC level ≥ 200 mg/dL, DM, FBG level ≥ 6.1 mmol/L, BMI ≥25 kg/m^2^, and Cr level > 1.2 mg/dL. Importantly, the consistent observation of the same U-shaped curve and an inflection point near 21.591 persisted across the GAM, smooth curve fitting, and recursive algorithm (Supplementary Figure S1).

**Table 6:** The outcomes derived from the two-piecewise linear regression model.

Unfavorable outcome	OR (95%CI)	*P*
Fitting model by standard linear regression	0.994 (0.973, 1.015)	0.569
Fitting model by two-piecewise linear regression Inflection point of the BUN/CR ratio	21.591	
≤21.591	0.942 (0.902, 0.985)	0.008
Per unit increase in Z score*	0.686 (0.519, 0.906)	
>21.591	1.055 (1.003, 1.110)	0.039
Per unit increase in Z score*	1.406 (1.018, 1.942)	
P for log-likelihood ratio test	<0.001	

We adjusted age, sex, WBC, RBC, MCHC, RDW, AST, ALT, FBG, FIB, TG, LDL-C, ALB, TP, GFR, BMI, DM, previous stroke or TIA, hypertension, AF, CHD, stroke etiology, smoking, and NIHSS score. *BUN/CR ratio was standardized with Z-transform (mean 0, SD 1) in each subgroup.

## 4 Discussion

In this prospective cohort study encompassing 1,906 Korean patients with AIS, the key findings can be succinctly outlined as follows. Firstly, the incidence of AOs at the 3-month mark post-AIS was 28.65%. Secondly, instead of a linear correlation, a U-shaped, nonlinear relationship was observed between the BUN/Cr ratio and outcomes at the 3-month juncture after AIS. Thirdly, a notable threshold effect manifested, pinpointing an inflection point at 21.591 for the BUN/Cr ratio. At this juncture, the incidence of AOs exhibited a linear increase when the BUN/Cr ratio veered toward either extreme, being exceptionally high or exceedingly low. Fourthly, a sensitivity analysis was meticulously executed, excluding individuals with TC ≥200 mg/dL, DM, FBG ≥6.1 mmol/L, BMI ≥25 kg/m^2^, and Cr >1.2 mg/dL. Following meticulous adjustment for potential confounding factors, the linear relationship between the BUN/Cr ratio and the risk of AOs remained statistically insignificant, affirming the persistence of a stable U-shaped nonlinear relationship. This study marks a significant contribution as the first of its kind to reveal a nonlinear U-shaped association between the BUN/Cr ratio and adverse 3-month outcomes following AIS. Although, the normal range of the BUN/Cr ratio is 10–20, the present study indicated that a BUN/Cr ratio closer to 21.591 is associated with a lower rate of adverse functional outcomes 3 months after AIS. Therefore, the BUN/Cr ratio may be a potential biomarker for predicting stroke prognosis. Consequently, optimizing the BUN/Cr ratio to approach 21.591 when managing patients with AIS may enhance their functional prognosis. This finding serves as a valuable foundation and guides the direction for our future research endeavors.

At present, the correlation between dehydration and stroke prognosis is a subject of contention. Some studies suggest that dehydration upon admission correlates with a heightened likelihood of severe disability or death among stroke cases. In a prospective cohort study involving 324 AIS cases, an increased BUN/Cr ratio was linked to an unfavorable 30-day prognosis (4). Another study, involving 126 participants, discovered that dehydration during stroke was associated with poorer short-term outcomes, as indicated by the change in the NIHSS score from admission to discharge (5). In another recent study involving 203 patients, the authors concluded that the BUN/Cr ratio is associated with larger volumes of ischemic tissue measured using diffusion-weighted imaging (DWI), and worse neurological deficits, as assessed by the NIHSS score, in AIS on admission (15). A broader exploration involving 3,059 AIS cases drawn from the Medical Information Mart for Intensive Care (MIMIC)-III and MIMIC-IV databases, with 2,085 cases subjected to propensity score matching (PSM) based on age and gender, indicated a slight association between the BUN/Cr ratio and an increased risk of in-hospital mortality in patients with AIS (RR = 1.01, 95% CI: 1.01, 1.02) (6). The disparities between these findings and those of the current study may be attributed to various factors. Firstly, the initial three studies had a limited sample size and a categorical dependent variable, dividing the BUN/Cr ratio into two groups with a cutoff of 15, in addition to different outcome variables. In contrast, our study employed the more authoritative mRS score at 3 months post-stroke as the outcome variable. Additionally, the present study included all cases with mild-to-severe AIS, making it more representative than studies exclusively focused on critically ill patients with AIS.

Nevertheless, subsequent to a MLRA, certain investigations have indicated that there is no discernible association between dehydration and AOs post-stroke, which closely align with the findings of the present study. In a study involving 3,355 cases, no statistically significant link was observed between an elevated BUN/Cr ratio and in-hospital all-cause mortality or AOs upon discharge (8). Another study, encompassing 5,971 participants, revealed that dehydration (defined as a BUN/Cr ratio > 20 or 30) at admission was correlated with stroke severity and the percentage of deaths at 3 months post-stroke, rather than indicating worse functional outcomes (7). In a cohort study involving 1,738 AIS cases from a Western Chinese population, the BUN/Cr ratio initially exhibited a positive association with 3-month outcomes (OR = 1.02, 95% CI: 1.00, 1.03, *p* = 0.04); however, this association lost significance after adjustments for potential confounders (*p* = 0.95) (9). Following GAM analysis, the nonlinear relationship between BUN/Cr and the 3-month outcomes was found. Using a two-piecewise regression model, the inflection point was identified as 18.78. This finding is similar to that of our study; however, whether on the left of the inflection point (OR = 0.98, 95% CI: 0.94, 1.02, *p* = 0.30) or the right side (OR = 1.01, 95% CI: 0.99, 1.03, *p* = 0.45), relationships between BUN/Cr-ratio and the 3-month mRS were not significant. In addition, this study also found that high HDL-C levels affected the relationship between the BUN/Cr-ratio and 3-month outcomes. However, the association between the BUN/Cr ratio and 3-month outcomes was not significant in patients with either low HDL-C levels or normal HDL-C levels. There was no interaction with HDL in our study (Supplementary Table S5). The difference between the results of this study and ours may be due to the slightly larger sample size of our study, which was 1,986 cases. In addition, although our study was derived from a secondary study on nutritional tools, the indicators collected were more comprehensive than those of the former. For example, we collected more indicators, such as BMI, WBC, RBC, HCT, HGB, MCHC, RDW, MCV, GRF, ALT, AST, FBG, FIB, APTT, stroke etiology, and history of coronary heart disease and previous stroke/TIA, than the previous study. Furthermore, we adjusted for more confounding factors, and the results were more reliable.

A high BUN/Cr ratio has been conventionally viewed as an indicator of dehydration, although the precise threshold remains uncertain, encompassing values greater than 15 (4, 5, 16), 20 (7), 25 (17, 18), or 30 (7). Dehydration emerges as a significant contributor to mortality, particularly in older patients (19). The ramifications of dehydration include diminished blood flow to muscles and kidneys, resulting in reduced cardiac output, thereby compromising the assurance of adequate oxygen delivery and normal organ function (4). Inadequate cerebral blood perfusion or collateral circulation may elevate the risk of stroke (18, 20), or accelerate stroke progression (12). Studies have posited that an elevated BUN/Cr ratio could serve as a potential marker for early neurological deterioration in AIS cases (21, 22). Furthermore, an elevated BUN/Cr ratio signifies serious medical conditions and an adverse prognosis in cases with acute kidney injury and acute heart failure (23, 24). Insights gained from modest-scale interventional trials have suggested that fluid regimens targeting the reduction of the BUN/Cr ratio in dehydrated patients with stroke upon admission may alleviate stroke exacerbations (25), enhance collateral cerebral perfusion (26), and curtail the length of the hospital stay (16).

An exceedingly low BUN/Cr ratio indicates a state of hyperhydration, leading to an augmented cardiovascular preload and elevated blood pressure. Consequently, this escalation in cardiovascular strain heightens the susceptibility to heart failure and stroke (27). A diminished BUN/Cr ratio is correlated with heightened risks of total incident stroke and ischemic stroke (20). Acute kidney injury is typically characterized by a sudden surge in serum creatinine levels, diminished urinary output, or a combination of both (28–30). A mounting body of evidence posits that the cardiovascular ramifications arising from acute kidney injury play a pivotal role in AOs (31), including an escalated risk of mortality and both short-term and long-term cardiovascular complications (32). In a comprehensive 2017 meta-analysis spanning 25 studies and encompassing 254,408 cases, of which 55,150 individuals experienced acute kidney injury, a notable 15% elevation in the risk of stroke over a 2.7-year period (IQR: 2.0, 3.4) was demonstrated (95% CI: 3, 28) (33). Some scholars regard kidney disease as a systemic condition that impacts numerous organs throughout the body, such as the heart, lung, liver and brain, and the kidney, and may initiate metabolic or humoral pathways that affect the function of distant organs (28). In elucidating the connection between a reduced BUN/Cr ratio and the initiation of stroke, potential underlying mechanisms may involve complex processes such as inflammation, activation of the renin–angiotensin–aldosterone system, stimulation of the renal sympathetic nervous system, oxidative stress, and endothelial dysfunction arising from acute renal injury (31). The rationale behind the puzzling U-shaped nonlinear relationship between the BUN/Cr ratio and an adverse prognosis 3 months following AIS remains elusive, necessitating a more comprehensive exploration through further investigative studies.

The current study presents several potential considerations or constraints. Firstly, its single-center focus and a study population consisting predominantly of Koreans limit the generalizability to other ethnic groups, necessitating additional research for validation of the findings. Secondly, the BUN/Cr ratio was evaluated solely at admission and was not reevaluated during the course of hospitalization, prompting the need for further exploration into potential variations over time. Thirdly, this was a secondary study, and the original study did not provide data on intravenous thrombolysis among the participants, nor did it clarify the specific reasons for the 72 patients who did not undergo the swallowing function test, which might have some influence on the results. However, the ratio of 72/2084 is very low, and the current rate of intravenous thrombolysis in patients with AIS worldwide, including Korea, is low; therefore, these factors are estimated to have little impact on the results.

## 5 Conclusion

In summary, this study revealed a non-linear U-shaped association between the BUN/Cr ratio and 3-month AOs in patients with AIS. When the BUN/Cr ratio fell below 21.591, a notably adverse correlation with AOs was observed. Conversely, when the BUN/Cr ratio exceeded 21.591, a significantly positive correlation with AOs emerged. Monitoring the BUN/Cr ratio may prove to be beneficial in identifying patients at a heightened risk of an unfavorable prognosis, providing substantial assistance to physicians in the management of such cases.

## Data availability statement

The raw data supporting the conclusions of this article will be made available by the authors, without undue reservation.

## Ethics statement

The studies involving humans were approved by the Institutional Review Board of Seoul National University Hospital. The studies were conducted in accordance with the local legislation and institutional requirements. The participants provided their written informed consent to participate in this study.

## Author contributions

HL: Writing – review & editing, Writing – original draft, Visualization, Methodology, Investigation, Formal analysis, Data curation, Conceptualization. YC: Writing – review & editing, Methodology, Investigation, Data curation. QZ: Writing – review & editing, Visualization, Formal analysis. GG: Writing – review & editing, Conceptualization. BH: Writing – review & editing, Writing – original draft, Methodology, Investigation, Data curation. FW: Writing – review & editing, Visualization, Formal analysis. JW: Writing – review & editing, Writing – original draft, Funding acquisition, Conceptualization.
