# Supplementary material for: Association between the blood urea nitrogen-to-creatinine ratio and 3-month outcomes in patients with acute ischemic stroke: a secondary analysis based on a prospective cohort study
Source: Front Neurol. 2024 Apr 17;15:1350116. doi: 10.3389/fneur.2024.1350116 (PMC11061497; doi:10.3389/fneur.2024.1350116)

***Supplementary*** ***Materials***

1. **Supplementary Figures and Tables**

**1.1 Supplementary Table**

**Supplementary Table 1.** Collinearity screening

|  | Step 1 | Step 2 | Step 3 |
| --- | --- | --- | --- |
| WBC | 1.2 | 1.2 | 1.2 |
| RBC | 208.9 | 161.9 | 2.2 |
| HB | 719.4 | 165.1 | NA |
| MCV | 27.5 | 21.1 | 1.4 |
| HCT | 801.2 | NA | NA |
| MCHC | 36.8 | 9 | 1.4 |
| RDW | 1.6 | 1.5 | 1.5 |
| PLT | 1.3 | 1.3 | 1.3 |
| TC | 5.4 | 5.4 | 5.4 |
| TG. | 1.5 | 1.5 | 1.5 |
| HDL-c | 1.6 | 1.6 | 1.6 |
| LDL-c | 4.3 | 4.3 | 4.3 |
| BUN | 5.6 | 5.5 | 5.5 |
| Cr | 3.7 | 3.7 | 3.7 |
| GFR | 2.8 | 2.8 | 2.8 |
| ALT | 2.2 | 2.2 | 2.2 |
| AST | 2.2 | 2.2 | 2.2 |
| ALB | 2.9 | 2.9 | 2.9 |
| TP | 2.2 | 2.2 | 2.2 |
| FBG | 1.4 | 1.4 | 1.4 |
| APTT | 1.1 | 1.1 | 1.1 |
| FIB | 1.3 | 1.3 | 1.3 |
| SEX | 2 | 2 | 2 |
| AGE | 1.5 | 1.5 | 1.5 |
| BMI | 1.3 | 1.3 | 1.3 |
| Previous stroke/TIA | 1.1 | 1.1 | 1.1 |
| Hypertension | 1.2 | 1.2 | 1.2 |
| DM | 1.4 | 1.4 | 1.4 |
| Smoking | 1.5 | 1.5 | 1.5 |
| AF | 1.3 | 1.3 | 1.3 |
| CHD | 1.1 | 1.1 | 1.1 |
| NIHSS score | 1.3 | 1.3 | 1.3 |
| Stroke etiology | 1.1 | 1.1 | 1.1 |

Selection method: Calculated VIF for each variable, if highest VIF value >= 5 , remove the variable with highest VIF;

Repeat the above step, until all remaining variables with VIF < 5

NA was the excluded variable

HGB, hemoglobin concentration; HCT, Hematocrit; MCV, mean corpuscular volume; PLT, platelet; TG, triglyceride; TC, total cholesterol; HDL-c, high-density lipoprotein cholesterol; LDL-c, low-density lipoproteins cholesterol; BUN, blood urea nitrogen; Scr, serum creatinine; ALT, alanine aminotransferase; AST, aspartate aminotransferase; ALB, serum albumin; FBG, fasting blood glucose; FIB, fibrinogen; BMI , body mass Index; DM, diabetes mellitus; CHD, coronary Heart Disease; TIA, transient ischemia attack . NIHSS, national Institute of health stroke scale; BUN/CR ratio, blood urea nitrogen to creatinine ratio.

**Supplementary Table 2.** Participants’ baseline characteristics categorized by the quintiles of the BUN/Cr ratio

| Characteristics | BUN/Cr-ratio(%) | | | | P |
| --- | --- | --- | --- | --- | --- |
|  | Q1(2.469-13.846) | Q2(13.861-17) | Q3(17.007-21.311) | Q4(21.333-58.621) |  |
| WBC(109/L) | 7.700(6.300-9.330) | 7.710(6.200-9.432) | 7.600(6.188-9.402) | 7.620(6.290-9.690) | 0.800 |
| RBC(1012/L) | 4.430(3.920-4.830) | 4.470(4.018-4.812) | 4.385(4.000-4.730) | 4.230(3.860-4.570) | <0.001 |
| HGB(g/dL) | 13.8(12.1-15.2) | 13.9(12.7-15.0) | 13.7(12.5-14.8) | 13.3(12.1-14.4) | <0.001 |
| HCT(%) | 41.1 (36.4-44.7) | 41.3 (37.775-44.425) | 40.6 (37.475-43.8) | 39.5 (36.2-42.5) | <0.001 |
| MCHC（%） | 33.70(33.00-34.40) | 33.75(32.90-34.40) | 33.60(32.80-34.30) | 33.50(32.80-34.30) | 0.420 |
| RDW（%） | 13.1(12.5-13.9) | 13.1(12.6-13.6) | 13.1(12.5-13.7) | 13.1(12.6-13.6) | 0.921 |
| MCV(fl) | 92.70(89.60-96.10) | 92.95(89.80-95.80) | 92.70(89.77-96.00) | 93.20(90.40-96.10) | 0.519 |
| PLT(109/L) | 217(186-262) | 216(182-256) | 215(176-257) | 219(177-260) | 0.471 |
| APTT（s） | 31.10 (28.90-33.30) | 30.70 (28.40-33.15) | 30.20(28.10-32.40) | 29.60(27.20-32.00) | <0.001 |
| FIB (mg/L) | 327 (280-372) | 320.50 (283.75-370.25) | 316.50 (277-366) | 321 (284-370) | 0.413 |

Values are mean ± standard deviation or median (quartile) or number (%), WBC white blood cell，RBC Red blood cell, HGB Hemoglobin concentration, HCT Hematocrit, MCV Mean corpuscular volume, MCHC mean corpuscular hemoglobin concentration, RDW Red blood cell distribution, PLT Platelet, FIB Fibrinogen, APTT Activated partial thromboplastin time

**Supplementary Table 3. Participants’ baseline characteristics categorized by 3 month outcomes.**

| Characteristics | Favorable outcome | Unfavorable outcome | P-value |
| --- | --- | --- | --- |
| participants  (%) | 1360(71.354%) | 546(28.646%) |  |
| Demographics |  |  |  |
| Age, years |  |  | <0.001 |
| ＜60 | 352 (25.882%) | 84 (15.385%) |  |
| 60 to 70 | 396 (29.118%) | 109 (19.963%) |  |
| 70 to 80 | 461 (33.897%) | 209 (38.278%) |  |
| ≥ 80 | 151 (11.103%) | 144 (26.374%) |  |
| Sex, n(%) |  |  | <0.001 |
| Male | 881 (64.779%) | 287 (52.564%) |  |
| Female | 479 (35.221%) | 259 (47.436%) |  |
| Smoking, n(%)  Medical history, n(%) | 580 (42.647%) | 170 (31.136%) | <0.001 |
| Hypertension | 838 (61.618%) | 373 (68.315%) | 0.006 |
| DM | 406 (29.853%) | 208 (38.095%) | <0.001 |
| CHD | 156 (11.471%) | 64 (11.722%) | 0.877 |
| AF | 242 (17.794%) | 165 (30.220%) | <0.001 |
| Previous stroke/TIA | 246 (18.088%) | 156 (28.571%) | <0.001 |
| Clincal features |  |  |  |
| BMI (kg/m2) | 23.758 ±3.125 | 22.853 ±3.474 | <0.001 |
| Baseline NIHSS score |  |  | <0.001 |
| ＜6 | 1161 (85.368%) | 216 (39.560%) |  |
| 6 to 13 | 141 (10.368%) | 167 (30.586%) |  |
| ≥14 | 58 (4.265%) | 163 (29.853%) |  |
| WBC(109/L) | 7.540 (6.172-9.270) | 8.000 (6.400-10.127) | <0.001 |
| RBC(1012/L) | 4.420 (4.020-4.772) | 4.240 (3.730-4.630) | <0.001 |
| HGB(g/dL) | 13.9 (12.6-14.9) | 13.15 (11.5-14.5) | <0.001 |
| HCT(%) | 41.1 (37.6-44.225) | 39.1 (34.8-43) | <0.001 |
| MCHC（%） | 33.7 (33-34.4) | 33.5 (32.7-34.3) | 0.003 |
| RDW（%） | 13.100 (12.500-13.600) | 13.250 (12.625-14.100) | <0.001 |
| MCV(fl) | 92.900 (90.075-95.925) | 92.800 (89.300-96.100) | 0.248 |
|  |  |  |  |
| PLT(109/L) | 217 (183.75-257) | 213.5 (171.25-260) | 0.069 |
| TC(mg/dl) | 180 (152-208) | 168 (142-202) | <0.001 |
| TG(mg/dl) | 102 (78-133.25) | 94 (71-115.75) | <0.001 |
| HDL-c(mg/dl) | 44.165 (38-53) | 44.165 (36-54) | 0.558 |
| LDL-c(mg/dl) | 105 (85-130) | 104.155 (77-128) | 0.025 |
| BUN(mg/dl) | 15 (13-19) | 16 (12-21) | 0.057 |
| Cr(mg/dl) | 0.900 (0.760-1.080) | 0.870 (0.700-1.107) | 0.092 |
| GFR（%） | 77.800 (63.375-93.200) | 77.500 (59.150-96.400) | 0.694 |
| ALT(U/L) | 23 (18-29) | 23 (18-31) | 0.076 |
| AST (U/L) | 18 (14-26) | 17 (12-25) | <0.001 |
| ALB (g/dl) | 4.1 (3.9-4.4) | 3.9 (3.6-4.2) | <0.001 |
| TP (g/dl) | 7.1 (6.7-7.4) | 6.9 (6.5-7.3) | <0.001 |
| FBG(mg/dl) | 98 (86-110) | 99.055 (89-123.75) | <0.001 |
| APTT（s） | 30.6 (28.4-32.8) | 30 (27.6-32.8) | 0.007 |
| FIB (mg/L) | 316 (278-361) | 332 (292.25-395.5) | <0.001 |
| BUN/Cr-ratio（%） | 16.949 (13.882-20.991) | 17.373 (13.846-22.658) | 0.042 |
| Ischemic stroke subtype |  |  | <0.001 |
| SVO | 443 (32.574%) | 163 (29.853%) |  |
| LAA | 297 (21.838%) | 68 (12.454%) |  |
| CE | 318 (23.382%) | 175 (32.051%) |  |
| Other determined | 97 (7.132%) | 74 (13.553%) |  |
| Undetermined | 205 (15.074%) | 66 (12.088%) |  |

WBC white blood cell，RBC Red blood cell, HGB Hemoglobin concentration, HCT Hematocrit, MCV Mean corpuscular volume, MCHC mean corpuscular hemoglobin concentration, RDW Red blood cell distribution, PLT Platelet, FIB Fibrinogen, APTT Activated partial thromboplastin time,BUN/Cr-ratio blood urea nitrogen to creatinine ratio, TG Triglyceride, TC Total cholesterol, HDL-c High-density lipoprotein cholesterol, LDL-c Low-density lipoproteins cholesterol, BUN Blood urea nitrogen, Cr Serum creatinine, GFR Glomerular filtration rate, ALT Alanine aminotransferase, AST Aspartate aminotransferase, ALB Serum albumin,TP Total protein, FBG Fasting blood glucose, BMI body mass index, DM Diabetes mellitus, CHD Coronary heart disease, AF Atrial fibrillation,TIA Transient ischemia attack. LAA Large artery atherosclerosis, SVO Small vessel occlusion, CE Cardio embolism, NIHSS National institute of health stroke scale.

**Supplementary Table 4.** Determinants of adverse outcomes in AIS assessed through univariate regression analysis

| Characteristics | OR 95%CI | P |
| --- | --- | --- |
| WBC(10^9^/L) | 1.079 (1.043, 1.116) | <0.001 |
| RBC(10^12^/L) | 0.583 (0.498, 0.682) | <0.001 |
| HGB(g/dL) | 0.819 (0.779, 0.862) | <0.001 |
| HCT(%) | 0.932 (0.916, 0.949) | <0.001 |
| MCHC（%） | 0.868 (0.797, 0.947) | 0.001 |
| RDW（%） | 1.232 (1.154, 1.316) | <0.001 |
| MCV(fl) | 0.988 (0.969, 1.007) | 0.219 |
| PLT(109/L) | 0.999 (0.998, 1.001) | 0.304 |
| APTT（s） | 0.988 (0.970, 1.007) | 0.205 |
| FIB (mg/L) | 1.003 (1.002, 1.004) | <0.001 |

WBC white blood cell，RBC Red blood cell, HGB Hemoglobin concentration, HCT Hematocrit, MCV Mean corpuscular volume, MCHC mean corpuscular hemoglobin concentration, RDW Red blood cell distribution, PLT Platelet, FIB Fibrinogen, APTT Activated partial thromboplastin time.

**Supplementary Table 5. subgroup analysis**

| Parameters | N(%) | OR | 95%CI Low | 95%CI High | P valu | P(interaction) |
| --- | --- | --- | --- | --- | --- | --- |
| Stroke etiology |  |  |  |  |  | 0.191 |
| SVO | 606 (31.794%) | 1.010 | 0.974 | 1.046 | 0.605 |  |
| LAA | 365 (19.150%) | 1.010 | 0.947 | 1.077 | 0.762 |  |
| CE | 493 (25.866%) | 1.022 | 0.981 | 1.063 | 0.297 |  |
| Other determined | 171 (8.972%) | 0.920 | 0.849 | 0.996 | 0.039 |  |
| Undetermined | 271 (14.218%) | 1.002 | 0.936 | 1.071 | 0.964 |  |
| HDL-c(mg/dl) |  |  |  |  |  | 0.338 |
| low (<1.09) |  | 1.120 | 0.830 | 1.511 | 0.460 |  |
| normal (1.09, 1.42) |  | 1.055 | 0.952 | 1.169 | 0.308 |  |
| high (≥1.42) |  | 0.991 | 0.970 | 1.012 | 0.401 |  |

Adjusted for age, sex, WBC,RBC,MCHC,RDW, AST,ALT,FBG,FIB, TG,LDL-C, ALB, TP, GFR,BMI,DM, previous stroke or TIA, hypertension,AF, CHD, stroke etiology, smoking, and NIHSS score

Note: In each stratification, the model was not adjusted for the stratification variable.

**1.2 Supplementary Figure 1. Sensitivity analyses.**


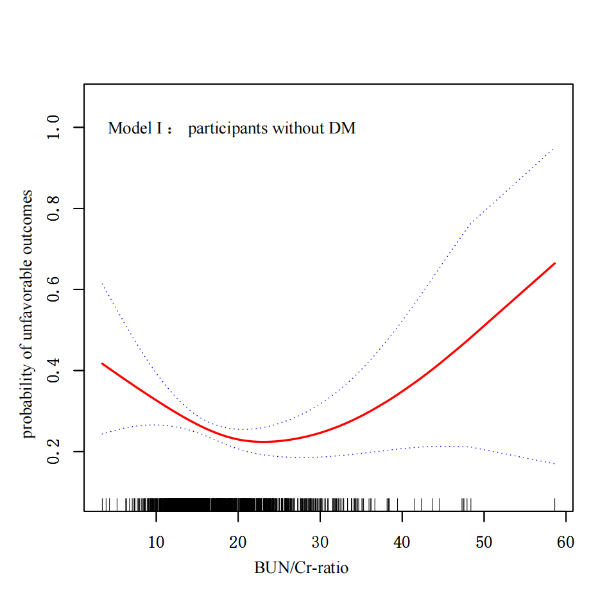

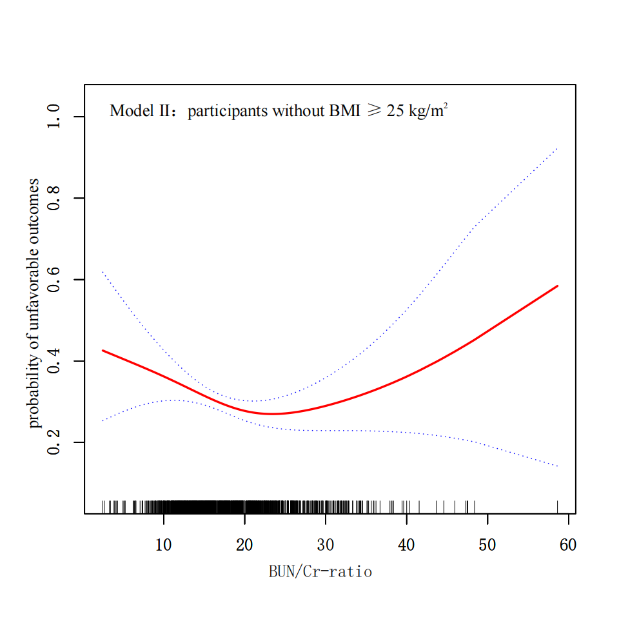


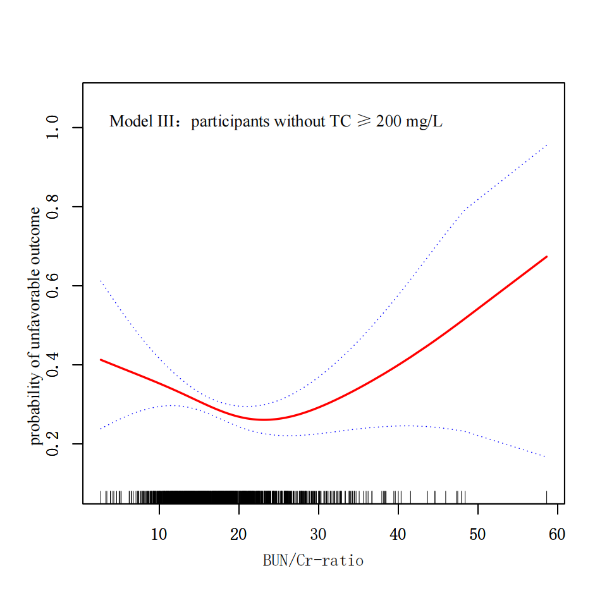

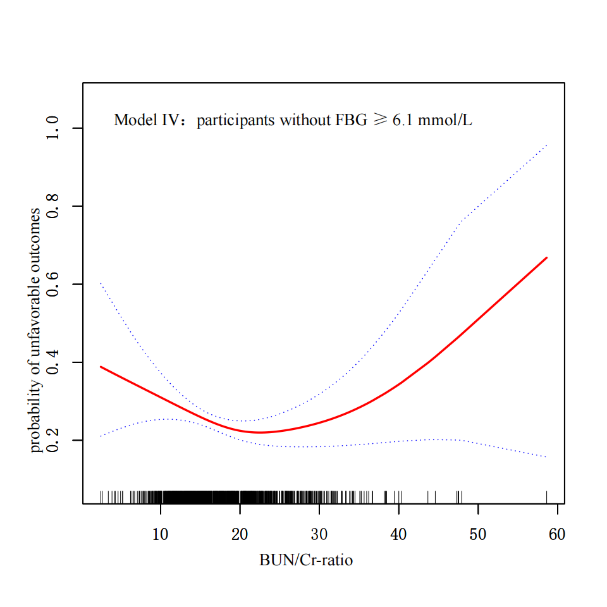


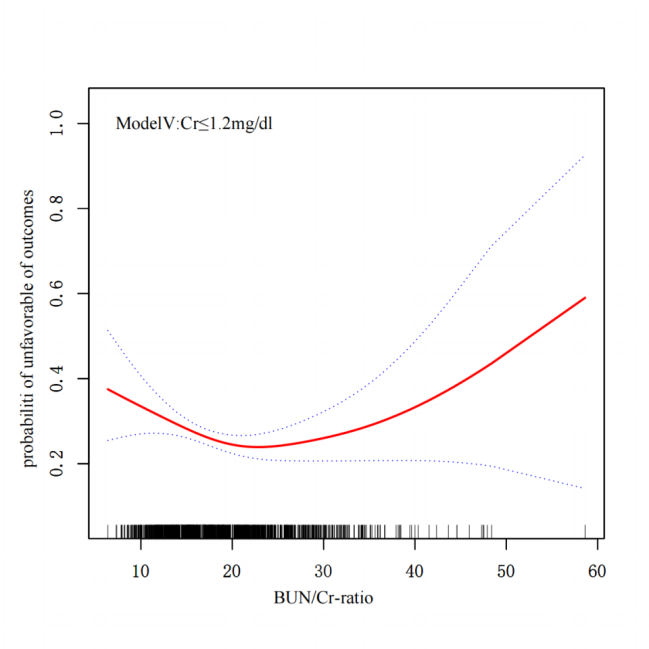

Supplement: Supplementary file 1 [file Data_Sheet_1.docx]
